# Generation of human otic neuronal organoids using pluripotent stem cells

**DOI:** 10.1111/cpr.13434

**Published:** 2023-02-24

**Authors:** Gaoying Sun, Mingming Tang, Xinyue Wang, Da Li, Wenwen Liu, Jianhuan Qi, Haibo Wang, Baoyang Hu

**Affiliations:** ^1^ State Key Laboratory of Stem Cell and Reproductive Biology, Institute of Zoology Chinese Academy of Sciences Beijing China; ^2^ University of Chinese Academy of Sciences Beijing China; ^3^ Department of Otolaryngology‐Head and Neck Surgery, Shandong Provincial ENT Hospital, Cheeloo College of Medicine Shandong University Jinan China; ^4^ Institute for Stem Cell and Regeneration Chinese Academy of Sciences Beijing China; ^5^ Beijing Institute for Stem Cell and Regenerative Medicine Beijing China; ^6^ National Stem Cell Resource Center Chinese Academy of Sciences Beijing China

## Abstract

Otic neurons, also known as spiral ganglion neurons (SGNs) in mammalian cochlea, transmit electrical signals from sensory hair cells to cochlear nuclei of the auditory system. SGNs are sensitive to toxic insults, vulnerable to get irreversible damaged and hardly regenerate after damage, causing persistent sensorineural hearing loss. Yet, to get authentic SGNs for research or therapeutic purpose remains challenging. Here we developed a protocol to generate human otic neuronal organoids (hONOs) from human pluripotent stem cells (hESCs), in which hESCs were step‐wisely induced to SGNs of the corresponding stages according to their developmental trajectory. The hONOs were enriched for SGN‐like cells at early stage, and for both neurons and astrocytes, Schwann cells or supporting cells thereafter. In these hONOs, we also determined the existence of typical Type I and Type II SGNs. Mature hONOs (at differentiation Day 60) formed neural network, featured by giant depolarizing potential (GDP)‐like events and rosette‐organized regions‐elicited calcium traces. Electrophysiological analysis confirmed the existence of glutamate‐responsive neurons in these hONOs. The otic neuronal organoids generated in this study provide an ideal model to study SGNs and related disorders, facilitating therapeutic development for sensorineural hearing loss.

## Graphical abstract

In the present study, we described a de novo 3D differentiation protocol to step‐wisely generate human otic neuronal organoids (hONOs) from human pluripotent stem cells (hESCs) according to the developmental trajectory of spiral ganglion neurons (SGNs). The hONOs were enriched in SGN‐like cells at early stage (bipolar), and glial cells at late stage (elongated dendrites expanding with neurites). Mature hONOs (at differentiation Day 60) formed neural network, evidenced by giant depolarizing potential (GDP)‐like events and rosette‐organized regions‐elicited calcium traces. Electrophysiological analysis confirmed the existence of glutamate‐responsive neurons in these hONOs. The otic neuronal organoids could be applied in the pathophysiological exploration of SGN‐involved disorders.

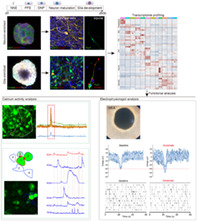

## 1 INTRODUCTION

Spiral ganglion neurons (SGNs) in mammalian cochlea transmit auditory signals from hair cells to cochlear nucleus in the central auditory system, which is essential for sound perception physiologically and pathologically. SGNs and hair cells are vulnerable to ototoxic insults including drugs, noise, genetic factors, ageing, and so on and the damage is permanent.^1^, ^2^ The fact is that the majority of sensorineural hearing loss is attributed to the irreversible damage of SGNs and/or hair cells and nowadays no curative treatments have been developed. Cochlear implants can partially replace the function of damaged hair cells depending on the existence of functional SGNs.^3^ While there are limited replacement or rescue therapies for SGNs dysfunction.

Efforts have been made to maintain and improve long‐term survival and function of cultured cells, especially peripheral neurons in vitro, included but not limited to the integration of bio‐materials, conductive substrates and induction of neurotrophic factors into the culture systems.^4^, ^5^, ^6^, ^7^, ^8^, ^9^, ^10^, ^11^, ^12^ Human SGNs are not well characterized along the developmental trajectory nowadays and the lack of suitable in vivo and in vitro experimental models might be one of the reasons. In recent years, the development of stem cell‐based technology has facilitated the generation of human otic lineages. The differentiation products containing SGNs function in different patterns. For SGNs targeted generation, Matsuoka et al. reported a step‐wise two‐dimensional (2D) differentiation protocol to generate SGN‐like cells from hESCs, and the differentiation observation was about 2 month with 20 days of neuronal maturation stage.^13^ The 3D organisation of differentiated cells has been recognized to better recapitulate in vivo counterparts compared with the 2D manner.^14^ For inner ear cells especially hair cells differentiation, Koehler et al. described a 3D chemically defined differentiation procedure, which finally produced well‐functioned hair cells from human pluripotent stem cells (hPSCs), and neurons were not further identified.^15^ Another differentiation protocol that generate inner ear cells was developed recently with a 2D combined with 3D method, which generate SGN‐like cells and hair cell‐like cells.^16^ SGN‐targeted otic organoids have not been developed yet.

The present study generated hPSC‐derived otic neuronal organoids (hONOs) harbouring SGN‐like cells and glial cells using a de novo 3D protocol without physical transition of formats after the initiation of differentiation (e.g., 2D to 3D). We identified that the hPSC‐derived aggregates went through non‐neural ectoderm (NNE), pre‐placodal ectoderm (PPE), otic neuronal progenitor (ONP), neuron maturation and gliogenesis, coinciding with the development trajectory of SGNs. Besides, at the late stage of hONO generation, glial cells present an explosive growth. Notably, hONOs formed neural network at early neuronal maturation stage. The hONOs generated in this study potentially facilitate the elucidation of physiological and pathological characteristics of SGNs in related research.

## 2 MATERIALS AND METHODS

### 2.1 hPSC lines and culture

**FIGURE 1:**
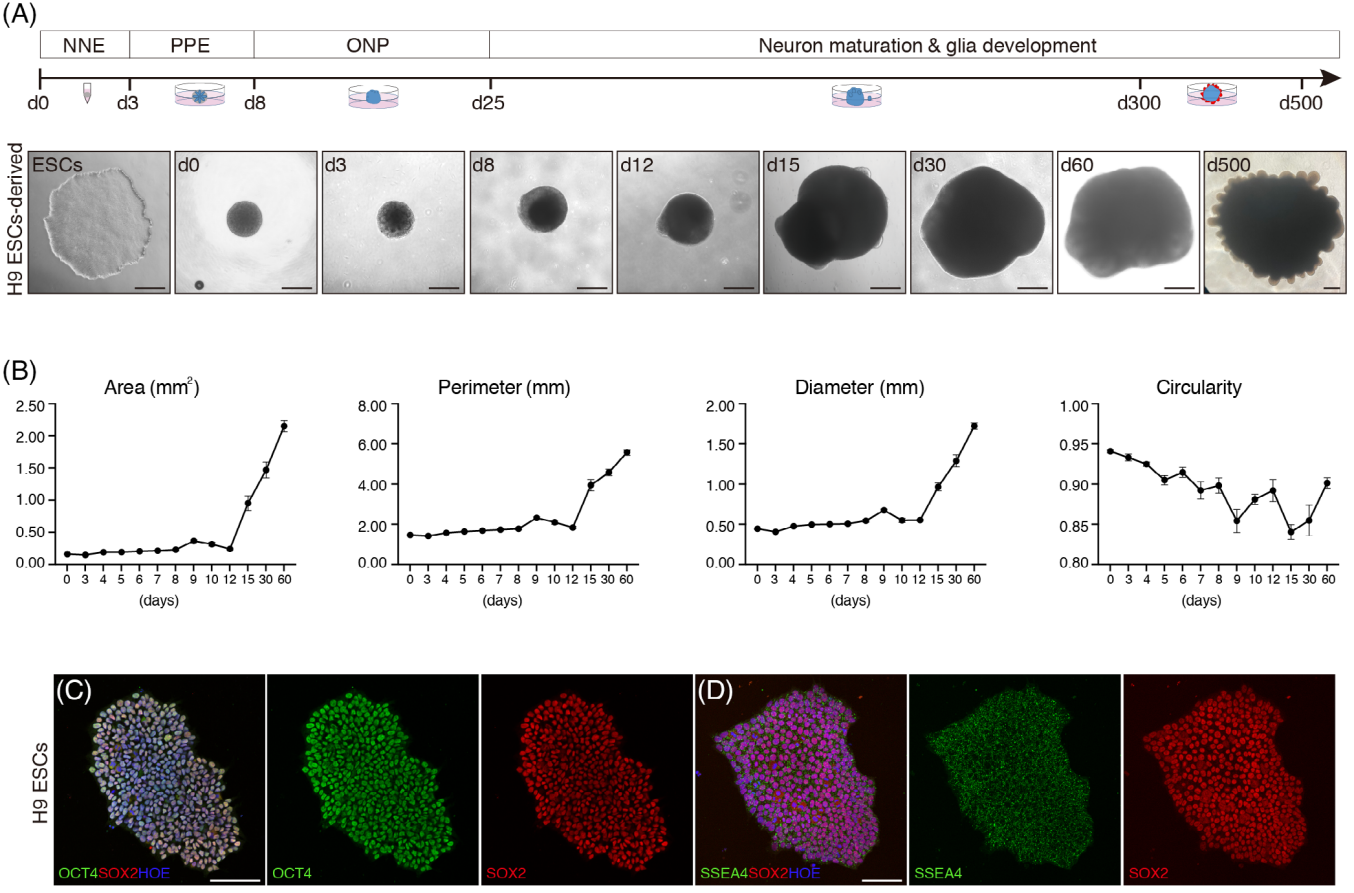
Schematic view showing the differentiation of human otic neuronal organoids (hONOs) from human pluripotent stem cells. (A) Differentiation strategy. Phase control images showing the dynamic changes of H9 ESC‐derived hONOs over time. Scale bars = 500 μm. (B) Analyses of morphology parameters of H9 ESCs‐derived aggregates over time, including area, perimeter, diameter and circularity. *n* = 5–61 organoids for each time point, at least three independent experiments. Data were shown as mean ± SEM. (C, D) Expression of pluripotency marker genes, OCT4, SOX2, and SSEA4, in monoclonal H9 ESCs. Scale bars = 100 μm.

hPSCs cell lines (H9 ESCs, passage 23–30; clinical‐grade Q‐CTS‐ESC‐2, CTS‐Q2 in brief in this study, passage 35–42) were used in this study. H9 ESCs were cultured on Matrigel (Corning)‐coated surface in Essential 8 (E8) medium (Invitrogen). CTS‐Q2 ESCs were maintained on human Vitronectin‐N (Invitrogen)‐coated surface in E8 medium. Both cell lines were passaged every 5–7 days using 0.5 mM EDTA. Before every series of differentiation, hPSCs were determined to be free of mycoplasma via a commercial detection kit (InvivoGen). Meanwhile, to reduce heterogeneity between series, pluripotency of initiated cells was determined via probing pluripotent marker genes, OCT4, SOX2, NANOG, and SSEA4 (Figures 1C,D and S1b–d) in monoclones.

### 2.2 Generation of hONOs

The step‐wise differentiation protocol of hONOs was made according to the developmental trajectory of SGNs reported previously (Figure 1A).^13^ To initiate differentiation, hESCs were dissociated into single cells using StemPro Accutase (Invitrogen) and seeded (3000 cells per well) into a low adhesive 96‐well plate (V‐bottom) in E8 medium supplemented with 10 μM Y27632 and 50 μg/mL Normocin (InvivoGen) at 37°C, 5% CO_2_. After 48 h of incubation, the well‐shaped aggregates (Figure 1A, differentiation Day 0, d0) were maintained in 96‐well V‐bottom plates in neural differentiation medium (NDM) supplemented with 10 ng/mL BMP4 (Peprotech), 10 ng/mL FGF2 (Peprotech) and 2 μM TGF‐β inhibitor SB431542 (Selleck) for 3 days. NDM contained a 50:50 mixture of Neurobasal medium (Gibco) and DMEM/F12 (Gibco) supplemented with 1X N_2_ (Gibco), 1X B27(Gibco), and 50 μg/mL Normocin. On d3, aggregates were pooled into a low adhesive 10‐cm culture dish on an orbital shaker in NDM containing 0.2 μM BMP inhibitor LDN193189 (Selleck), 2 μM SB431542, 4 μM Wnt pathway inhibitor IWP2 (Selleck) and 10 ng/mL FGF2 (Peprotech). On d8, the medium was changed to NDM supplemented with 3 μM CHIR99021, a Wnt pathway activator via inhibiting GSK3β, 10 ng/mL FGF2 and 50 ng/mL IGF1 (Peprotech). After 7 days of incubation, 0.5 μg/mL sonic hedgehog (SHH; Peprotech), 1 μM all‐trans retinoic acid (ATRA; Selleck), 20 ng/mL EGF (Peprotech), 10 ng/mL FGF2, and 50 ng/mL IGF1 were added to new NDM. On d23, SHH and ATRA were removed and 1 μM Y27632 was added. Between d25 and d30, medium was changed to 10 μM dibutyryl‐cyclic AMP (cAMP, Sigma), 20 ng/mL BDNF (Peprotech), 20 ng/mL NT3 (Peprotech), 20 ng/mL IGF1, and 1 μM Y27632 added to neural induction medium (NIM), that is, Neurobasal medium containing 1X N_2_, 1X B27, 1X GlutaMAX (Gibco) and 50 μg/mL Normocin. From d30 on, aggregates were bathed in NIM supplemented with 10 μM dibutyryl‐cAMP (Sigma), 20 ng/mL BDNF (Peprotech), 20 ng/mL NT3 (Peprotech), 20 ng/mL IGF1, 20 ng/mL GDNF (Peprotech), and 20 ng/mL CNTF (Peprotech). Shake incubation was changed to stationery pattern on d90.

### 2.3 Immunohistochemistry immunofluorescence

Immunohistochemistry monolayer‐cultured cells and 14‐μm cyosections were immersed in 4% paraformaldehyde (PFA) for 15 min at room temperature. After three washes with PBS, samples were blocked in 5% bovine serum albumin (BSA; Sigma), 1% donkey serum and 0.1 Triton X‐100 (Sigma) in PBS, naming block solution 1, for 1 h at room temperature. Cells were incubated in block solution 1‐diluted primary antibodies at 4°C overnight (see Table S1 for details). After three washes of PBS with 0.1% Triton X‐100 in PBS at room temperature for 2 h, samples were incubated in block solution 1‐diluted secondary antibodies (Invitrogen; 1:1000) and Hoechst 33342 (Sigma; 2 μg/mL) at room temperature for 90 min followed by 1‐h wash with PBS at room temperature. Immunostaining images were captured via a confocal microscope (ZEISS LSM 880).

### 2.4 Whole‐mount immunofluorescence

hONOs at selected time points were collected and fixed in 4% PFA for 48 h at 4°C followed by immersing in PBS for 6–8 h at room temperature. The following procedures of immunostaining were all performed at 4°C. hONOs were blocked in block solution 2 (5% BSA, 3% donkey serum, and 1% Triton X‐100 in PBS) for 8 h and incubated with block solution 2‐diluted primary antibodies for 2–5 days (depending on sizes of hONOs). Then samples were incubated with corresponding secondary antibodies as well as Hoechst 33342 diluted in PBS for 4–8 h (depending on the size of samples), followed by washing in PBS for 12 h. Whole‐mount immunostaining samples were imaged using the Z‐stack option in ZEN 2010 software installed on a ZEISS LSM 880 confocal microscope.

### 2.5 Morphological subtyping of neurons and glial cells in mature hONOs

To determine the morphology and quantification of neurons and glial cells in well‐functioned hONOs, samples were dissociated in Accutase at 37°C for 10 min, centrifuged at 2000 rpm for 5 min, re‐suspended and cultured for additional 24 h under 2D condition. Then monolayer‐cultured cells were fixed for the following immunochemistry procedures. Neurons were labelled using TUJ1, glial cells using S100B, and supporting cells using SOX2 primary antibodies. Cell counting was performed using ImageJ software.

### 2.6 Bulk RNA sequencing

For RNA sequencing (RNA‐seq), aggregates were collected on different days of differentiation, d0, d3, d8, d15, d25, d30, d60, d90, d150, d300, d400, and d500. For early stage (d0–d8), each sample contained three to seven organoids, and from d15 and on, each sample contained only one organoid. Extraction of RNA was performed using TRIzol method (Invitrogen) and quality of RNA was determined using a commercial assay kit (Agilent Technologies). For each sample, 0.1 μg RNA was used for generation of cDNA libraries via VAHTS Universal V6 RNA‐seq Library Prep Kit for Illumina (NR604‐01/02) according to the manufacturer's instructions. Sequencing was performed on the Novaseq 6000 S4 platform. Gene expression was represented by fragments per kilo base per million (FPKM) values and a gene with FPKM value >1 was considered to be expressed in detected samples. The transcriptome sequencing and analysis was conducted by Xiuyue Biol. Principal component analyses (PCA), correlation heatmap, dot analysis, hierarchical heatmap, differentially expressed genes (DEGs), and Gene Oncology (GO) analyses were performed to assess gene expression patterns.

### 2.7 Calcium imaging

To evaluate the calcium activity, whole hONOs at d60 were labelled with 1 μM Fluo‐4AM in NIM at 37°C for 1 h according to the recommended instructions. Then calcium fluorescence for each sample was visualized on a ZEISS LSM 880 inverted confocal microscope at room temperature no more than 2 h. Fluo‐4AM was excited at 488 nm and time‐lapse images were captured every 5–10 s. Glutamatergic neurons were activated by 10 μM glutamic acid (Sigma) for 5 min at room temperature. Calcium responses were quantified by relative fluorescence intensity (Δ*F*/*F*) and Δ*F*/*F* value ≥0.4 was considered to be calcium‐responsive.

### 2.8 Multi‐electrode array assay

Single‐well plates with 256 TiN electrodes (spacing 200 μm and diameter 30 μm; Multichannel Systems) were used to assess neural network developed in d60 hONOs. After hONOs were mounted on 1% Matrigel‐precoated multi‐electrode array (MEA) well and cultured for 72 h, MEA recording was performed on a MEA2100 system using the equipped Multi Channel Experimenter software (Multichannel systems). To assess the glutamate reactivity of hONOs, 10 μM glutamic acid (Sigma) was added to NIM and incubated at 37°C for 2 min. Recordings were analysed using Multi Channel Analyser software and electrodes detected at least 5 spikes/min were considered as active.

### 2.9 Statistical analysis

hONOs were performed at least for three differentiation series derived from H9 and CTS‐Q2 ESCs. Data were shown as mean ± SEM. Statistical analysis was performed on Prism 9 GraphPad software using two‐tailed unpaired *t*‐test for two groups or one‐way ANOVA using Dunnett's multiple comparison for three and more groups, which were indicated in corresponding figure legends. *p* < 0.05 was assumed to be statistically significant.

## 3 RESULTS

### 3.1 Step‐wise induction of NNE‐ and PPE‐like cells

**FIGURE 2:**
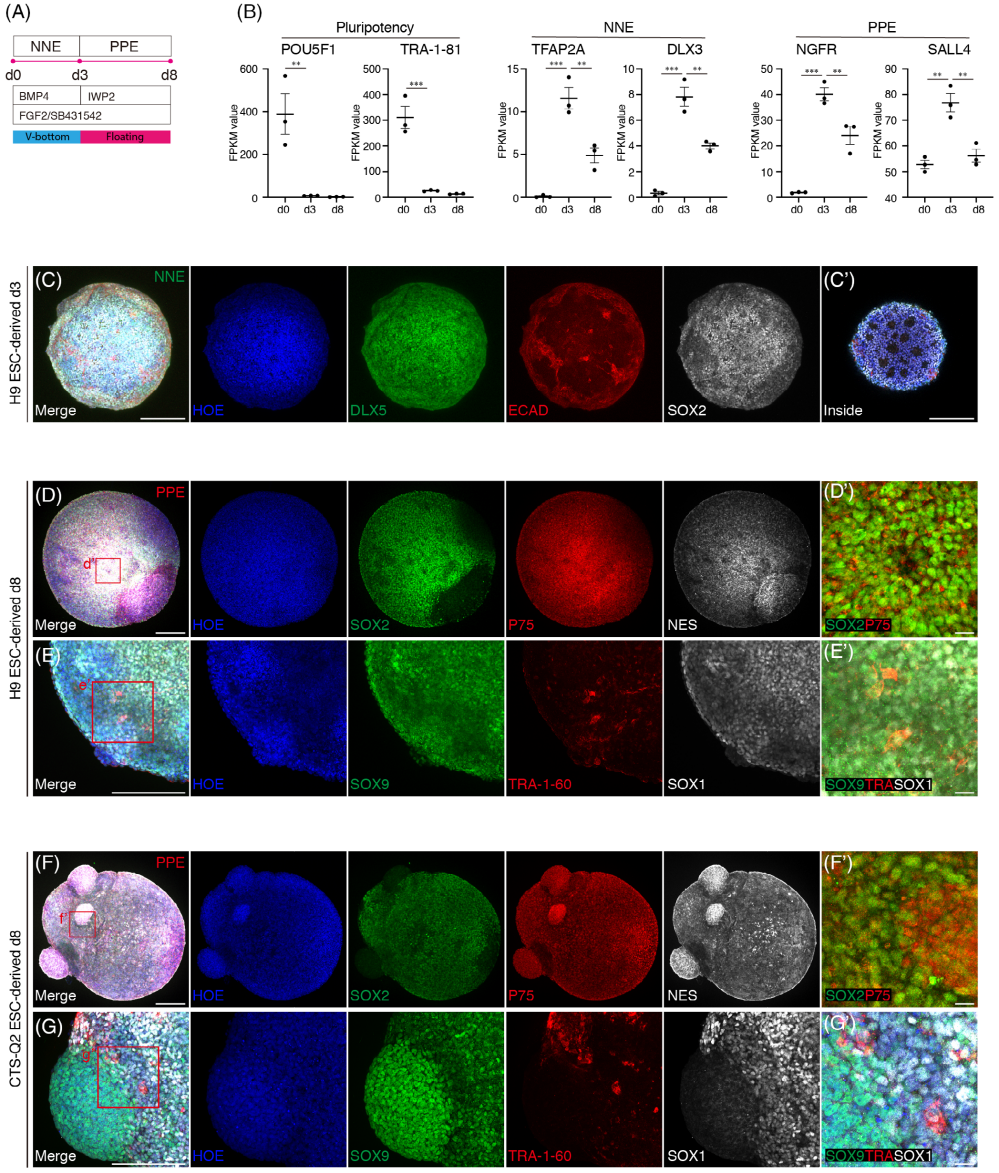
Induction and characterization of NNE‐ and PPE‐like cells from human pluripotent stem cells. (A) Differentiation strategy of NNE and PPE induction. (B) Expression of pluripotency, NNE, and PPE marker genes during d0 to d8. *n* = 3–7 organoids for each sample, two independent experiments. Data were shown as mean ± SEM. **p < 0.01; ***p < 0.001. (C) Expression of NNE markers (DLX5, ECAD) in d3 H9 ESC‐derived organoids. C′ showing the image inside of this organoid. Scale bars = 200 μm. (D, E) Expression of PPE marker (P75) and pluripotency marker (TRA‐1‐60) in d8 H9 ESC‐derived organoids. SOX9, marker of glial cell. SOX1, marker of neuronal progenitor. Scale bars = 200 μm (overview), 20 μm (close‐up). (F, G) Identification f PPE marker (F) and pluripotency maker in d8 CTS‐Q2 ESC‐derived organoids. Scale bars = 200 μm (overview), 20 μm (close‐up).

Previous studies illustrated that BMP activation and TGF‐β inhibition promised efficient NNE induction of hPSCs in 2D^13^ and 3D differentiation patterns.^15^ After 3 days of NNE induction, expression of NNE marker genes TFAP2A and DLX3 in aggregates were significantly increased with neglectable expression of pluripotency marker genes, suggesting cells at this stage might be NNE‐like cells (Figure 2A,B). Extensive existence of DLX5‐positive (NNE marker) cells in d3 aggregates indicated the efficient conversion to NNE stage (Figure 2C). P75 (also known as NGFR) is commonly used as positive sorting for otic epithelium in 2D differentiation of SGNs. PPE marker NGFR and SALL4 increased early at NNE stage and decreased on d8, even though the expression was still at high level (Figure 2B). In d8 hONOs derived from H9 ESCs, high ratio of P75‐positive cells across whole aggregates guaranteed the acquisition of otic epithelium at this stage (Figure 2D). A small amount of TRA‐1‐60‐positive cells in d8 hONOs indicated the loss of pluripotency of hPSCs at PPE stage. Considerable amount of SOX1‐positive cells (marker for neuronal progenitor) in d8 hONOs suggested that some cells already differentiated into neuronal progenitors (Figure 2E). In addition, SOX9‐positive cells in d8 hONOs symbolized the co‐development of glia cells as early as PPE stage (Figure 2E). The similar expression profiles of related genes were recapitulated in CTS‐Q2 ESC‐derived hONOs (d8), proving the applicability of NNE‐PPE differentiation in this study (Figure 2F,G).

### 3.2 Identification of ONPs

**FIGURE 3:**
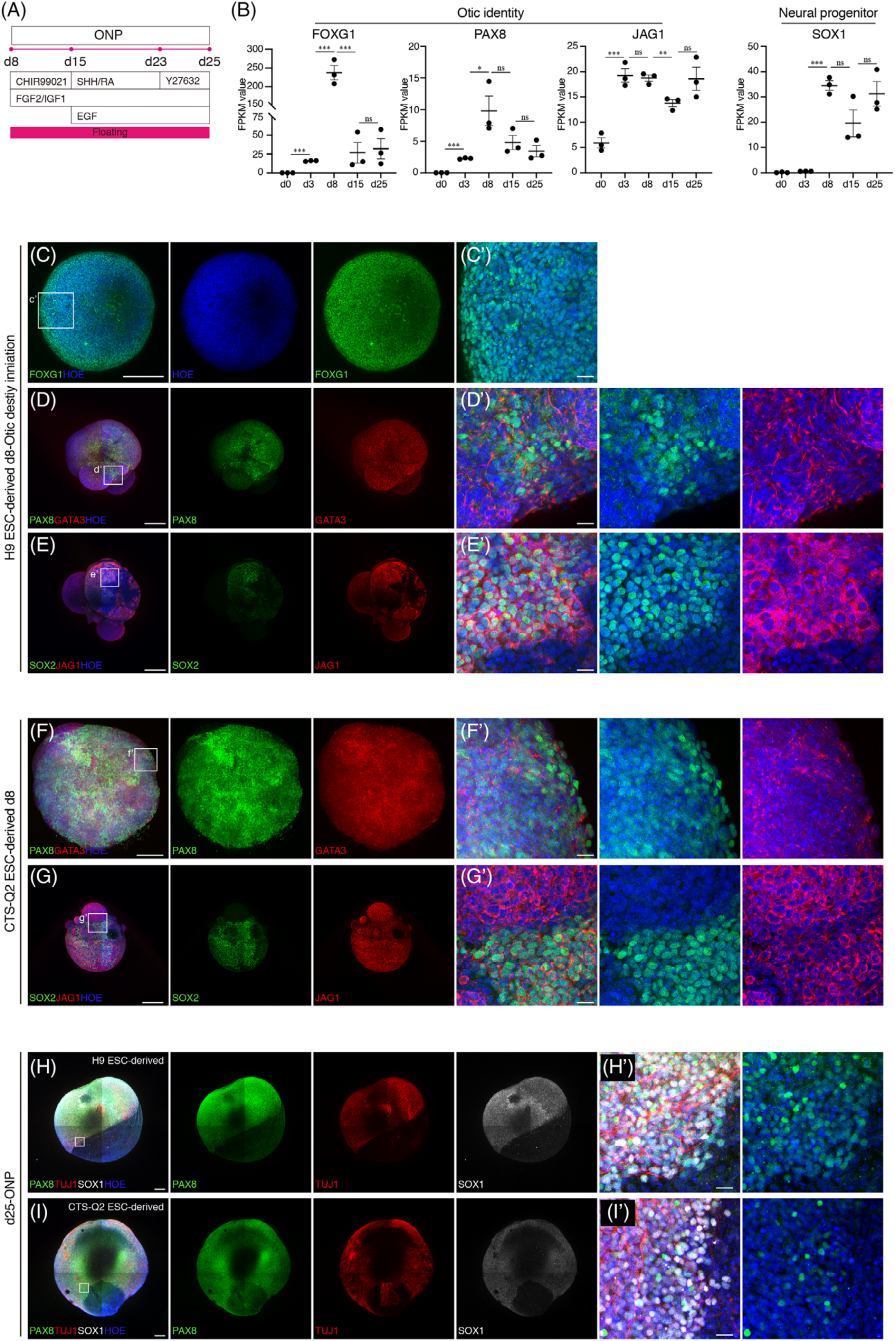
Initiation and maintenance of otic identity for hONOs derived from human pluripotent stem cells. (A) Differentiation strategy of ONP induction. (B) Expression of otic and neural progenitor marker genes during d0–d25. *n* = 1–3 organoids for each sample, two independent experiments. Data were shown as mean ± SEM. Two‐tailed unpaired *t*‐test. *p < 0.05; **p < 0.01; ***p < 0.001. ns, no significance. (C–G) Representative images identifying the otic destination initiation of H9 ESC‐ (C–E) and CTS‐Q2 ESC‐derived (F, G) hONOs on d8. Otic markers, FOXG1, GATA3, PAX8. Scale bars = 200 μm (overview), 20 μm (close‐up). (H, I) Representative images of otic neural progenitor markers, PAX8 and SOX1, in H9 ESC‐ (H) and CTS‐Q2 ESC‐derived (I) hONOs on d25. Scale bars = 200 μm (overview), 20 μm (close‐up).

The developmental stage of SGNs after PPE is ONP. At the end of PPE induction (d8), some SOX1‐positive cells emerged and expression of SOX1 significantly increased compared with that on d0, which reminded us to further make sure the identity of this cell population at this stage. Astonishingly, expression of FOXG1 and PAX8 (otic markers), was the highest between d0 to d25 (Figure 3B). And expression of JAG1, another otic marker, was kept at high level from d3 to d25 (Figure 3B). These transcriptome results suggested us that ONP stage might start from d8. The positive staining of otic markers (FOXG1, PAX8, GATA3, and JAG1) in d8 hONOs derived from H9 and CTS‐Q2 ESCs substantiated the initiation of otic destination from d8 (Figure 3C–G). On d25, a relatively large amount of ONPs (PAX8 for otic identity, SOX1 and TUJ1 for neuronal progenitor) declared that ONP stage might not terminate at once (Figure 3H,I).

### 3.3 Neurons in hONOs resemble SGNs cellularly and morphologically

**FIGURE 4:**
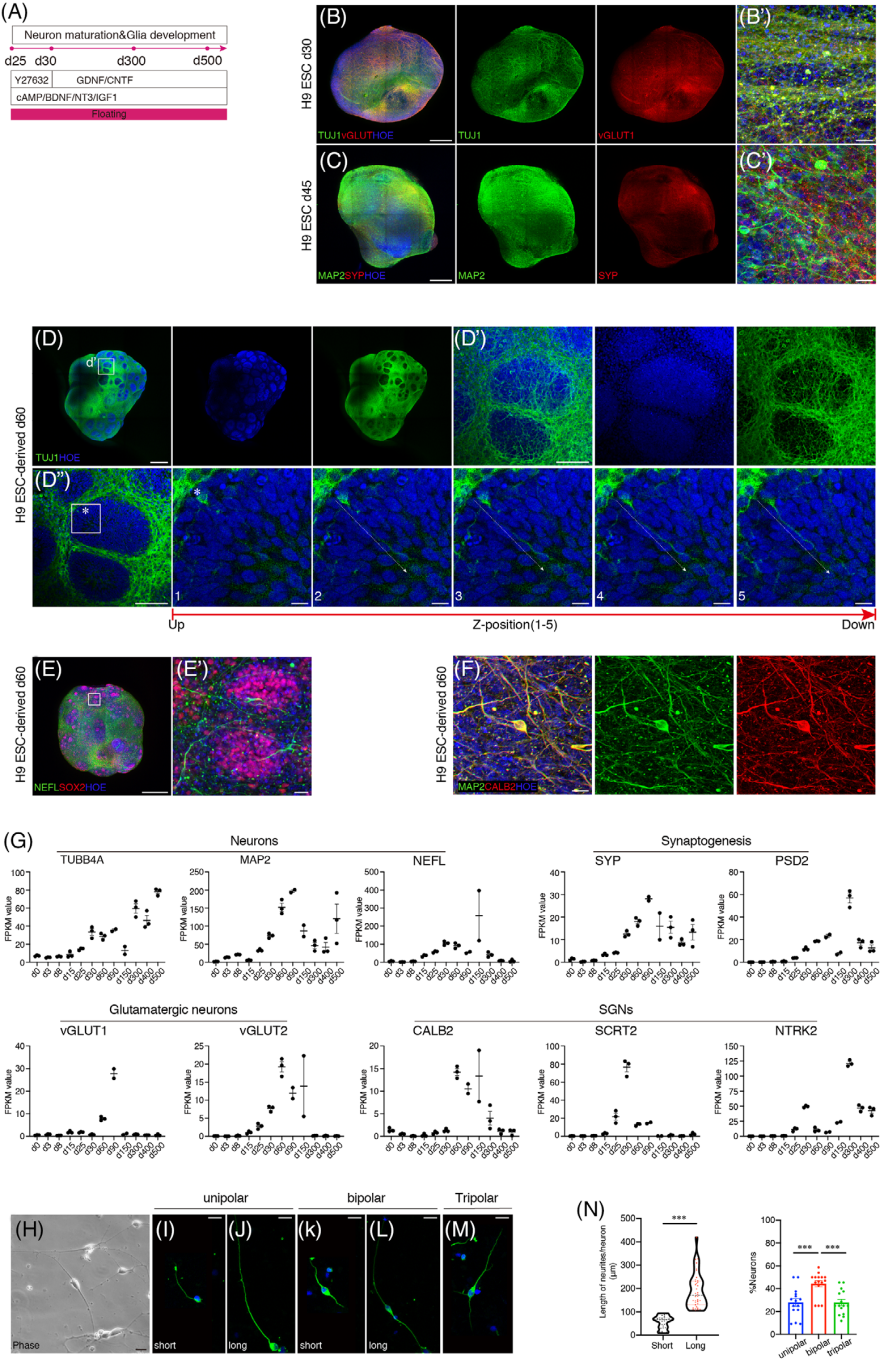
Identification of SGN‐like cells on the surface of mature hONOs. (A) Differentiation strategy after ONP induction. (B, C) Whole‐mount immunostaining of H9 ESC‐derived hONOs on d30 (B) and d45 (C) to identify the existence of glutamatergic neurons (vGLUT1) and mature functional neurons (MAP2, SYP). (D) The overview image of neural clusters (TUJ1+ wrapped) in d60 hONOs. (D′) Close‐up view showing the neuron connection between and on the surface of clusters. (D″) Illustrating the neurite extension of asterisk‐labelled neuron inside the cluster. (E) Expression of neural axon marker gene NEFL in d60 hONOs. (F) Expression of SGN marker gene CALB2 in mature neurons (MAP2+) in d60 hONOs. (G) Expression of marker genes of neurons, synaptogenesis, glutamatergic neuron, and SGNs during d0–d500. *n* = 1–3 organoids for each sample, two independent experiments. Data were shown as mean ± SEM. (H–M) Neurons from dissociated hONOs at d90. (N) Quantification of neurons in d90 hONOs for neurite length and subtyping. *n* = 3 organoids, two independent experiments. Two‐tailed unpaired *t*‐test. ***p < 0.001. Scale bars = 500 μm (B–E), 200 μm (D′, D″), 20 μm (B′, C′, Z1–Z5, E′, F, H).

Neuronal progenitors needed to differentiate into mature neurons for physical performances (Figure 4A). On d30 (5 days after ONP induction), hONOs were wrapped by TUJ1‐positive neurites and puncta expressing were detected along and among neurons, indicating the existence of glutamatergic neurons (Figure 4B). On d45, mature neurons (MAP2‐positive) emerged across the whole surface of hONOs with puncta expression of synaptic protein synaptophysin (SYP) along neurons (Figure 4c). On d60, specific structure, neuron‐wrapped clusters were observed in the majority of hONOs, where neurons (TUJ1‐positive) formed nets among clusters and extended into the inside of cluster (Figure 4D). At this stage, peripheral neuron marker NEFL was expressed extensively across the surface of hONOs and around SOX2‐positive clusters (Figures 4E and S2a). Most importantly, SGN‐specific marker CALB2 co‐expressed with MAP2‐positive neurons in d60 hONOs (Figures 4F and S2c). These above results indicated that d60 hONOs maintained mature SGN‐like cells. Systematic evaluation of genes about mature neurons, synaptogenesis, glutamatergic neurons, and SGN identity gave out the conclusion that d60–d150 might be the stage when neurons function well (Figure 4G). Cells dissociated from d90 hONOs were cultured in monolayer pattern to observe the morphology of neurons. Neurons were classified according to polarity and neurite length, where half neurons were bipolar and length of neurites between short and long neurites is significantly different (Figure 4H–N).

### 3.4 Glia prosperity at the late stage of hONOs generation

**FIGURE 5:**
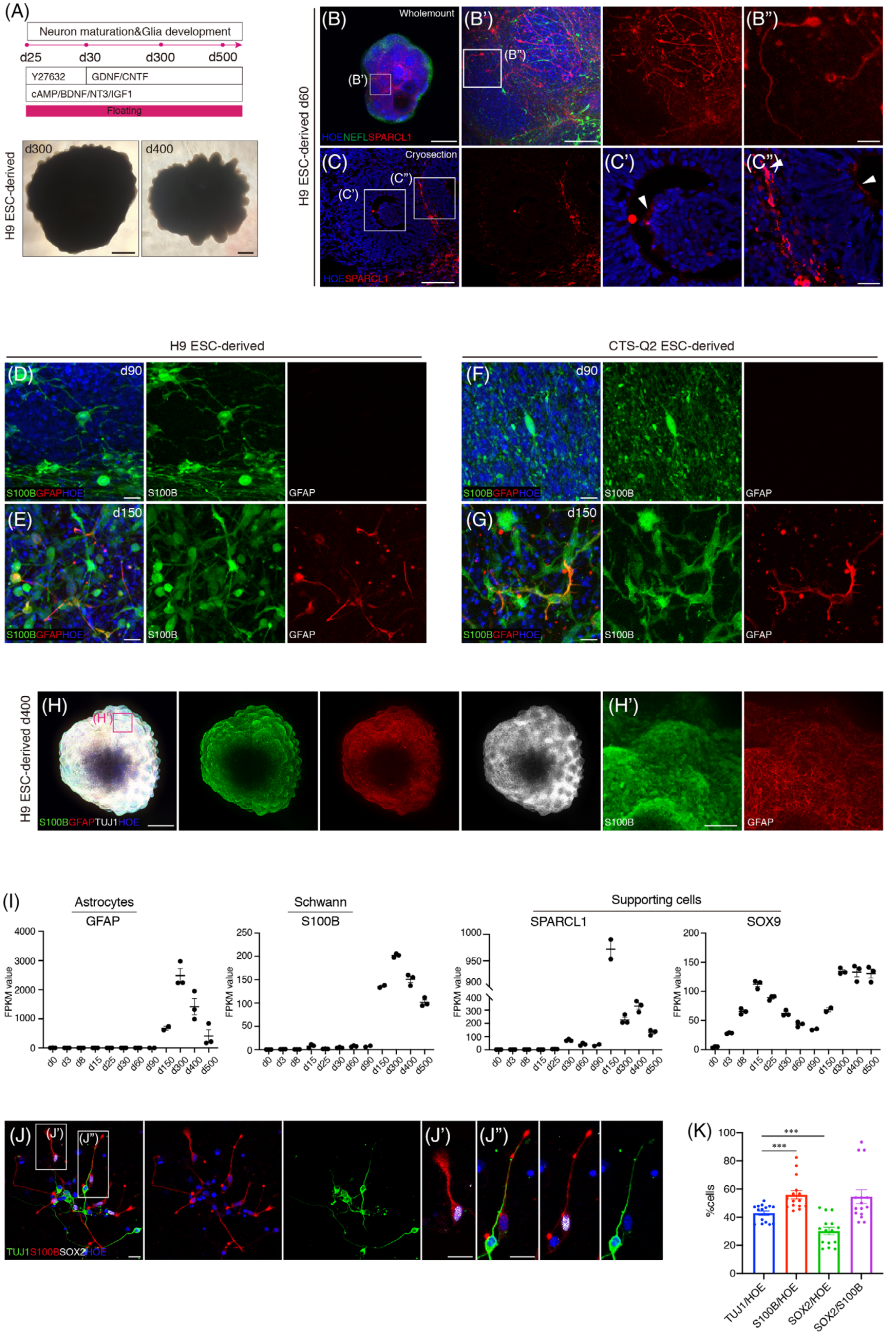
Glial lineages boom at the late phase of hONOs. (A) Differentiation strategy after ONP induction. (B, C) Whole‐mount (B) and cryosection (C) immunostaining of d60 hONOs to illustrate the existence and location of supporting cells (SPARCL1). (D–G) Representative images of schwann cells (S100B) and astrocytes (GFAP) in H9 ESC‐derived (D, E) and CTS‐Q2 ESC‐derived hONOs between d90 and d150. (H) Extensive existence of schwann cells (S100B) and astrocytes (GFAP) in d400 hONOs. (I) Expression of marker genes of different kinds of glial cells, astrocytes, schwann cells as well as supporting cells in hONOs from d0 to d500. *n* = 1–7 organoids for each sample, two independent experiments. Data were shown as mean ± SEM. (J) Neurons (TUJ1) and glial cells (S100B, SOX2) from dissociated hONOs derived from H9 ESCs (d90). (K) Quantification of neurons and glial cells in dissociated hONOs derived from H9 ESCs (d90). *n* = 3 organoids, two independent experiments. Data were shown as mean ± SEM. Two‐tailed unpaired *t*‐test. ***p < 0.001. Scale bars = 500 μm (B, H), 100 μm (B′, C, H′), 20 μm (B″, C′, C″, D, E, F, G, J).

Bright field observation of hONOs showed that on d300 and afterwards, hONOs presented many protrusions at the border (Figures 1A, 5A, and S1a). Considering that mature neurons do not proliferate, we hypothesized that hONOs might contain non‐neuronal lineages, highly possible including glia cells. Supporting cells (SPARCL1‐positive) were found to be located around and inside the SOX2‐positive clusters accompanied with NEFL‐positive neurons (Figures 5B and S2b). On d90, Schwann cells (S100B‐positive) were began to be detected and surged on d150, and astrocytes (GFAP‐positive) were first determined on d150 (Figure 5). The protrusions of d400 hONOs were proved to be mainly composed of Schwann cells and astrocytes with neurons with truncated neurites (Figure 5H). Expression of glial cells, including astrocytes, Schwann cells and supporting cells, mainly matched with the immunostaining results. Morphological analysis of neurons and glial cells of hONOs on d90 indicated that the ratio of neurons was a little bit lower than that of glial cells (Figure 5J,K). In addition, S100B‐positive glial cells shaped depending on the accompany of neurons or not, where glial cells with neuron co‐existence extended long dendrites and single glial cell had short and inflated dendrites (Figure 5J).

### 3.5 Transcriptome identification of pattern profiles during hONO generation

**FIGURE 6:**
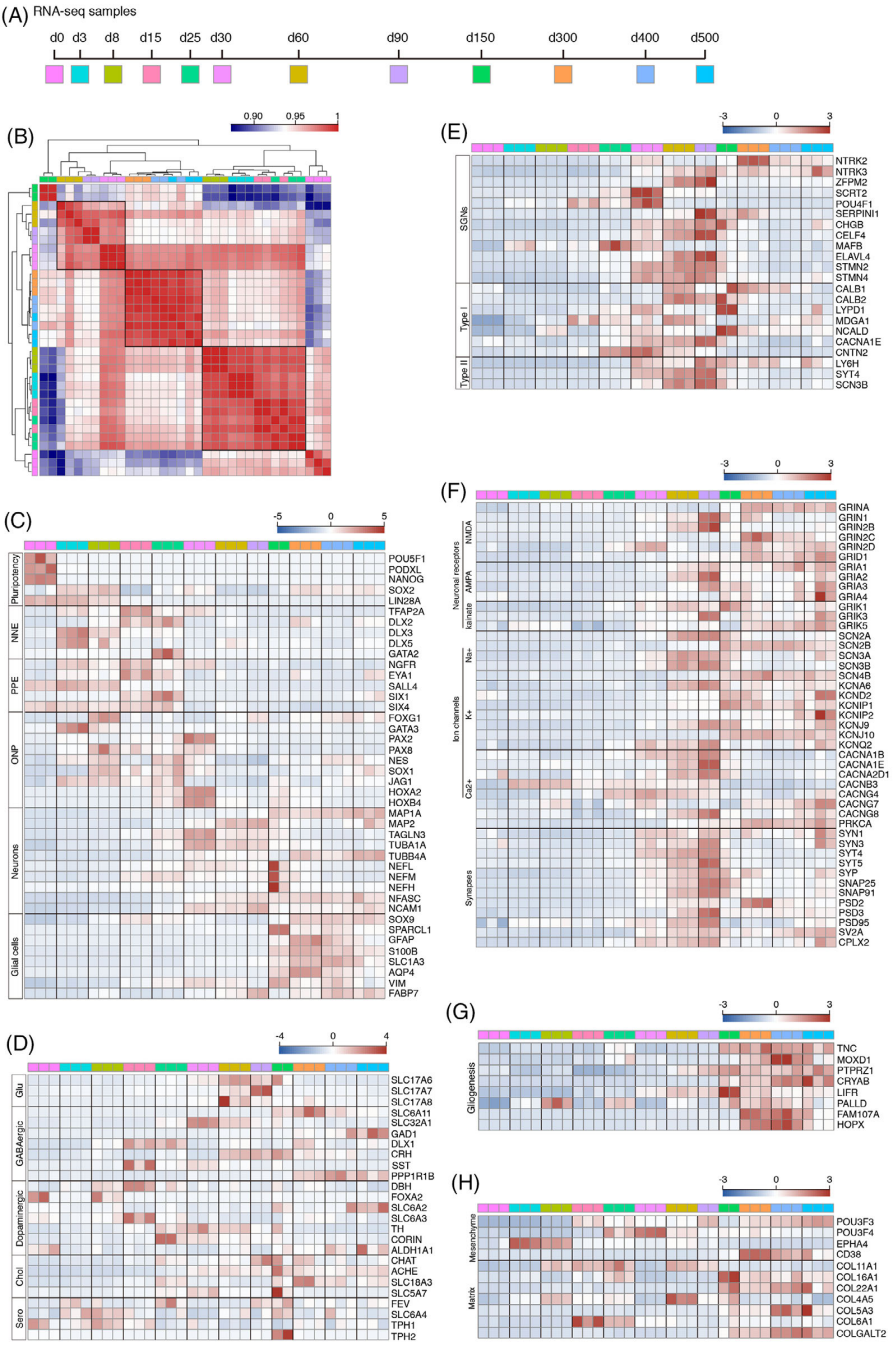
Transcriptome profiling during hONO generation by RNA sequencing. (A) Timepoints for RNA sample collection. (B) Correlation heat map illustrating data clustering of hONOs at different differentiation days. (C) Heat map showing expression of marker genes for distinct developmental phases: pluripotency, non‐neural ectoderm (NNE), pre‐placodal ectoderm (PPE), otic neuronal progenitor (ONP), neurogenesis, and gliogenesis. (D) Heatmap showing transcripts encoding for glutamatergic (Glu), GABAergic, dopaminergic, cholinergic (Chol) and serotonergic (Sero) neurons in hONOs. (E) Expression of common SGN marker genes and subtyping marker genes. (F) Heat map showing transcripts related with synaptic transmission, including glutamatergic neuronal receptors, ion channels and synaptic proteins. (G) Transcripts for gliogenesis marker genes. (H) Heatmap showing gene expression related with mesenchymal cells and matrix components.

To evaluate the differentiation profiling, we performed bulk RNA‐seq of hONOs sampled on different time points, from d0 to d500 (Figure 6A). PCA and cluster analysis indicated the phase overlap of d60–d90, d3–d25, d300–400 (Figures 6B and S3). Vast expression of pluripotent markers characterized the stem cell in d0 aggregates. NNE and PPE marker genes were highly expressed from d3 to d25, and ONP marker genes highly expressed until d30. Most neuronal marker genes were enriched on d30–d150 and glia cells on d150–d500 (Figure 6C). On d60–d150, markers of different kind of neurons (especially glutamatergic neurons) and SGN‐specific markers as well as subtyping markers, were enriched expressed (Figure 6D,E). From d60 and on, markers of glutamatergic receptors as well as neuronal function‐related genes were enriched identified (Figure 6F). hONOs at d150–d500 featured as enrichment of genes related with gliogenesis which might partially explain the prosperity of glial cells at this stage (Figure 6G). From d25 and on, markers of otic mesenchymal cells were enriched and markers of matrix component were enriched from d150 and on (Figure 6H). To summarize, hONOs are featured as neurogenesis during d60–d150, and as gliogenesis after d150. As the morphology of hONOs changed distinctly, we hypothesized that cellular composition as well as function of hONOs at this time point were completely different from the former time points. We compared the transcriptome profiles of hONOs from d300 with that from d60. Compared with d60, 7449 genes were differently expressed in hONOs of d300 (Figure S3b,c). In d300 hONOs, 49.68% of DEGs are upregulated. GO analysis enriched upregulated DEGs in biological process (GO.BP) and cellular components (GO.CC) in d300 hONOs, revealing that GO terms related with the regulation of neuron function and gliogenesis were enriched in d300 (Figure S3d,e).

### 3.6 Neuronal network is well constructed in hONOs

**FIGURE 7:**
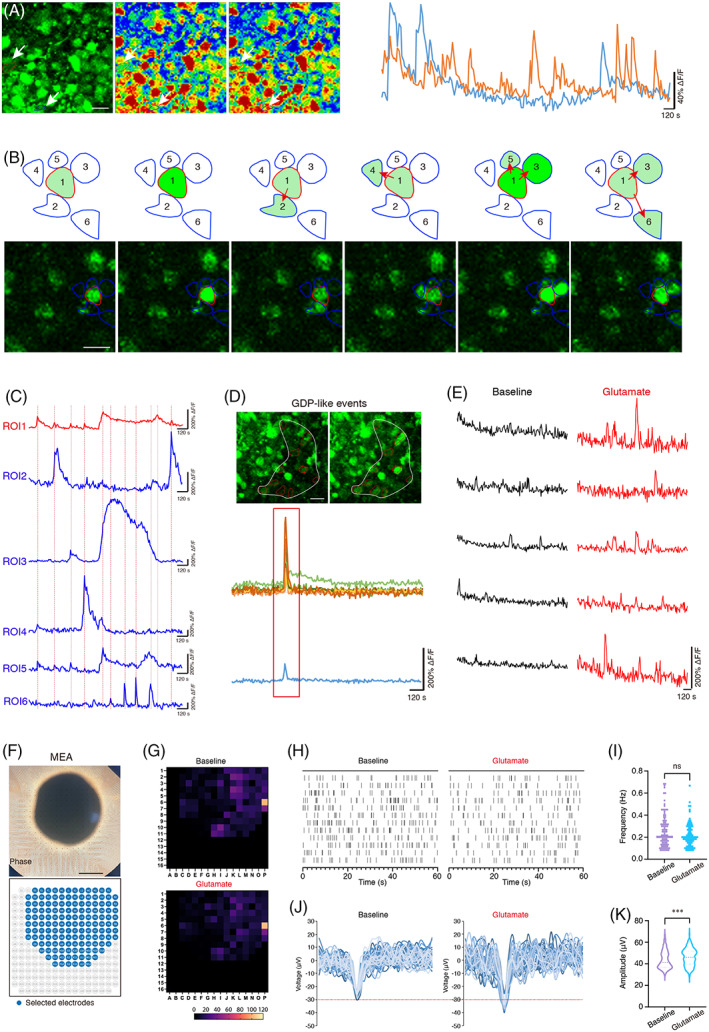
Neural network is developed in hONOs. (A) Calcium activity in d60 hONOs derived from H9 ESCs. Left: overview of the calcium‐responsive regions in hONOs. Middle: Heatmaps of selected regions of interest (ROIs). Right: Calcium traces of selected two ROIs. (B) Rosette‐like organized calcium‐responsive regions. Time‐lapsed images of circled ROIs indicating correlation of calcium‐response between core ROI (1) and peripheral ROIs (2–5). (C) Calcium traces of rosette‐like organized ROIs in (B). (D) Calcium activity and CTRs showing GDP‐like events. (E) Calcium traces of glutamate‐responsive ROIs. (F) Bright field image of 256 MEA with a whole hONO from d60. Bottom: Selected electrodes according to loading position of hONO. (G) Heat maps according to spike frequency under baseline and glutamate stimulation. (H) Representative traces of d60 hONO under baseline and glutamate stimulation. (I) Quantification of spike frequency. (J) Representative waves of spikes collected. (K) Quantification of spike amplitude. *n* = 4 organoids, three independent experiments. Data were shown as mean ± SEM. Two‐tailed unpaired *t*‐test. ***p < 0.001. ns, no significance. Scale bars = 500 μm (F), 20 μm (A–C).

The above results revealed that hONOs (d60–d150) might mainly focused on neuronal function. Next we confirmed the existence of functional neurons and neural network in d60 hONOs via calcium imaging and MEA. There were extensive calcium‐responsive regions in spontaneous hONOs, suggesting the presence of functional neurons (Figure 7A; Video S1). Interestingly, we found many rosette‐like calcium‐responsive regions under spontaneous conditions (Figure 7B; Video S2). The time‐lapse imaging showed a population of calcium traces (CTRs) that fired in a specific rhythm, that is, the core region fired to peripheral regions at different directions sequentially (Figure 7B). The CTRs fired in core region (ROI1) were similarly appeared in peripheral regions (Figures 7C and S4a and S4c; Videos S2 and S3), closely resembling the transduction of neural signal between two neurons in vivo. We also detected giant depolarizing potential (GDP)‐like events in hONOs, that is highly organized and synchronous calcium responses in isolated regions, indicating the formation of neural network (Figure 7D and S4b; Video S1). As SGNs belong to glutamatergic neurons, we found a large number of glutamate‐responsive regions after glutamate stimulation with different phenotypes, including increased frequency and/or Δ*F*/*F* value (Figure 7E), indicating the containing glutamatergic neurons functioned dimensionally, possible matched with different types of SGNs. MEA analyses were performed to monitor the spatiotemporal neuronal network (Figure 7F). Glutamate stimulation did not affect the frequency of spikes detected on active electrodes (Figure 7G–I), while the amplitude of spikes detected was significantly increased compared with the baseline (Figure 7J,K), further proving that the glutamatergic neurons might be in different types for distinct reactivity to glutamate, which is consistent with the results of calcium activity analysis.

## 4 DISCUSSION

Until recently, the elucidation of SGNs development and degeneration was hindered due to the lack of human SGNs materials. The present study described a de novo 3D differentiation protocol that differentiates hESCs into otic neuronal organoids majoring in SGN‐like cells. According to the developmental trajectory of SGNs in mammalian inner ear, NNE is the beginning of otic induction.^15^ Then NNE develops into PPE and SGN precursors.^17^ We sequentially identified NNE, PPE, ONP marker genes expressed in hONOs at corresponding differentiation time points, indicating that the final neurons belong to otic lineages.

Mammalian SGNs in the cochlea contains two basic types, where 95% are Type I SGNs that connect to inner hair cells and 5% are Type II SGNs that connect to outer hair cells.^18^, ^19^ Type I SGNs are all bipolar neurons and connect with one inner hair cell. Type I SGNs differ in sound sensitivity and spontaneous electrical activity.^20^, ^21^, ^22^, ^23^ Type II SGNs are pseudounipolar neurons and extend long neurites to receive signals from multiple outer hair cells.^24^ Previous studies illustrate that Type II SGNs play critic roles in noise‐induced sound sensation.^25^, ^26^ In this study, half of neurons in mature hONOs were bipolar, proving the SGN identity from the aspect of morphology. In the transcriptome analysis, we found the enriched expression of SGN marker genes as well as markers specific for Type I and Type II SGNs.

Glial cells are shown to be essential to the survival, neural network, and refinement of neurons.^27^ Zafeiriou et al. reported the facilitation of glial cells to neuronal maturation in brain organoids.^28^ In the mammalian inner ear, there are various types of glial cells, such as Schwann cells, satellite glial cells, supporting cells, astrocytes and so on.^29^, ^30^ In previous inner cell differentiation protocols, gliogenesis‐related growth factors were seldom induced in order to produce more hair cells and neurons.^15^, ^16^ In our study, to generate sufficient glial cells, we added cytokine CNTF to the medium, which is reported to promote astrogliogenesis and produce astrocytic progenitors from neural stem cells, at the early maturation of neurons.^31^ To our expectations, diversified glial lineages, including supporting cells, astrocytes and Schwann cells were enriched in hONOs at late differentiation stage. In the morphologic analysis of glial cells and neurons, some S100B^+^SOX2^+^ glial cells extended dendrites along with neurites, suggesting that this type of glial cells might served as Schwann cells, which are related with the myelination of SGNs.^15^ On the other hand, the short and expanded dendrites of another kind of S100B^+^SOX2^+^ cells did not go with neurites, indicating that they might be supporting cells. The enrichment of glial lineages makes hONOs be a useful source for glia‐related studies about inner ear disorders.

Synaptogenesis is acknowledged to compromise the neuron maturation.^32^, ^33^ In our study, we determined that synaptogenesis was initiated at d30, and continued to increase until d150, which reminded us that neurons at this stage might form connectivity and exert plasticity. We detected morphological neural network at d60 and calcium activity analysis gave out more solid results about the construction of neural network, including GDP‐like events and rosette‐organized regions‐elicited CTRs. GDP events have been reported to be commonly detected in murine and human brain, marking the development of neural network.^34^, ^35^ Interestingly, we found a new pattern of calcium responses in spontaneous condition, that is, the rosette‐organized regions‐elicited CTRs. We found the core ROI of rosette manipulated the pattern of CTRs elicited by peripheral ROIs, resembling the signal transduction between neighbour neurons. This phenomenon consistently appeared in all detected hONOs at d60–d90 (*n* = 4). Further exploration of this phenomenon would be performed in hONOs at all time points. In addition, glutamate responses were educed in mature hONOs evidenced by enhanced frequency and amplitude in consistent with glutamate‐triggered performances of murine SGNs explants as previously described.^36^ The differentiation protocol was also reproduced using a clinic‐grade pluripotent stem cells which added up to its applicability for clinical requirements.

In the present study, we did not characterize SGN subtypes using molecular markers which needed to be completed in the future study. Besides, we only determined the neural function of hONOs at limited time points, and those at the early and late stages are not mentioned. The further functional analyses including connection with hair cells and cochlear nucleus as well as drug responses needed to be performed.

## 5 CONCLUSIONS

The present study described a de novo 3D differentiation protocol that differentiates hESCs into otic neuronal organoids majoring in SGN‐like cells at early stage and glial cells at late stage. The existence of unipolar and bipolar neurons as well as transcriptomic identification for SGN subtypes marker genes added up to the SGN identity of neurons in hONOs. In addition, neural network was well constructed in hONOs. These SGN‐like cells in hONOs could provide a source for functional neuron replacement in sensorineural hearing loss as well as glial lineage‐targeted studies in inner ear field.

## AUTHOR CONTRIBUTIONS

**Gaoying Sun:** Designed the project, performed the experiments, analysed data, and wrote the manuscript. **Mingming Tang:** Analysed MEA data. **Xinyue Wang:** Collected raw data of calcium imaging, assisted the conduction of immunostaining and electrophysiological experiments. **Da Li:** Guaranteed the material preparation and normal functioning of experimental instruments. **Jianhuan Qi** and **Wenwen Liu:** Supplied with constructive suggestions during the manuscript writing. **Haibo Wang:** Supervised the project. **Baoyang Hu:** Conceived, designed, and supervised the project, and drafted the manuscript.

## CONFLICT OF INTEREST STATEMENT

The authors declare no conflict of interest.

## Supporting information


**FIGURE S1.** Schematic view of CTS‐Q2 ESC‐derived human otic neuronal organoids. a, Phase control images showing the changes of CTS‐Q2 ESCs‐derived hONOs over time. Red dashed line showing the growth curve. b, Representative image of CTS‐Q2 ESC clones maintained on VTN‐coated surface in E8 medium. c, d, Expression of pluripotency marker genes, OCT4, SOX2, NANOG and SSEA4, in monoclonal CTS‐Q2 ESCs. Scale bars, 500 μm (a), 200 μm (b), 100 μm (c, d).

**FIGURE S2.** SGN‐like cells and supporting cells appear in CTS‐Q2 derived hONOs at d60. a, Expression of axon marker gene (NEFL) and supporting cell marker gene (SOX2) in d60 hONOs derived from CTS‐Q2 ESCs. vGLUT1‐positive cells indicating the existence of glutamatergic neurons. b, Supporting cells (SPARCL1+) companied with axons (NEFL) in d60 hONOs. c, Expression of SGN‐specific marker gene (CALB2) and mature neuron marker gene (MAP2) in d60 hONOs. Scale bars, 500 μm (a), 100 μm (a′), 20 μm (b, c).

**FIGURE S3.** Transcriptome analysis of hONOs (d0‐d500). a, Principal component analysis (PCA) depicting similarity between hONOs at the same timepoints. b, Cluster analysis of differentially expressed genes (DEGs) via comparing d300 with d60 hONOs. c, Pie chart showing up‐ and downregulated DEGs in d300 hONOs compared with d60 hONOs. d, Enriched GO.BP (d) and GO.CC (e) items in d300 hONOs compared with d60 hONOs.

**FIGURE S4.** Neural network in hONO at neuronal stage. a, Heat map of rosette ROIs indicated in Figure 7B. b, Calcium traces of individual ROI that contributed to a GDP‐like event in Figure 7D. c, Calcium traces of another rosette‐like population of ROIs that resembles neighbour neuron communication.

**TABLE S1.** Antibodies

**VIDEO S1.** Supporting Information

**VIDEO S2.** Supporting Information

**VIDEO S3.** Supporting Information

## Data Availability

The supporting data of this study are only available from the corresponding author based on reasonable requirements.
