# Supplementary figures and images for: Generation of human otic neuronal organoids using pluripotent stem cells
Source: Cell Prolif. 2023 Feb 24;56(5):e13434. doi: 10.1111/cpr.13434 (PMC10212712; doi:10.1111/cpr.13434)

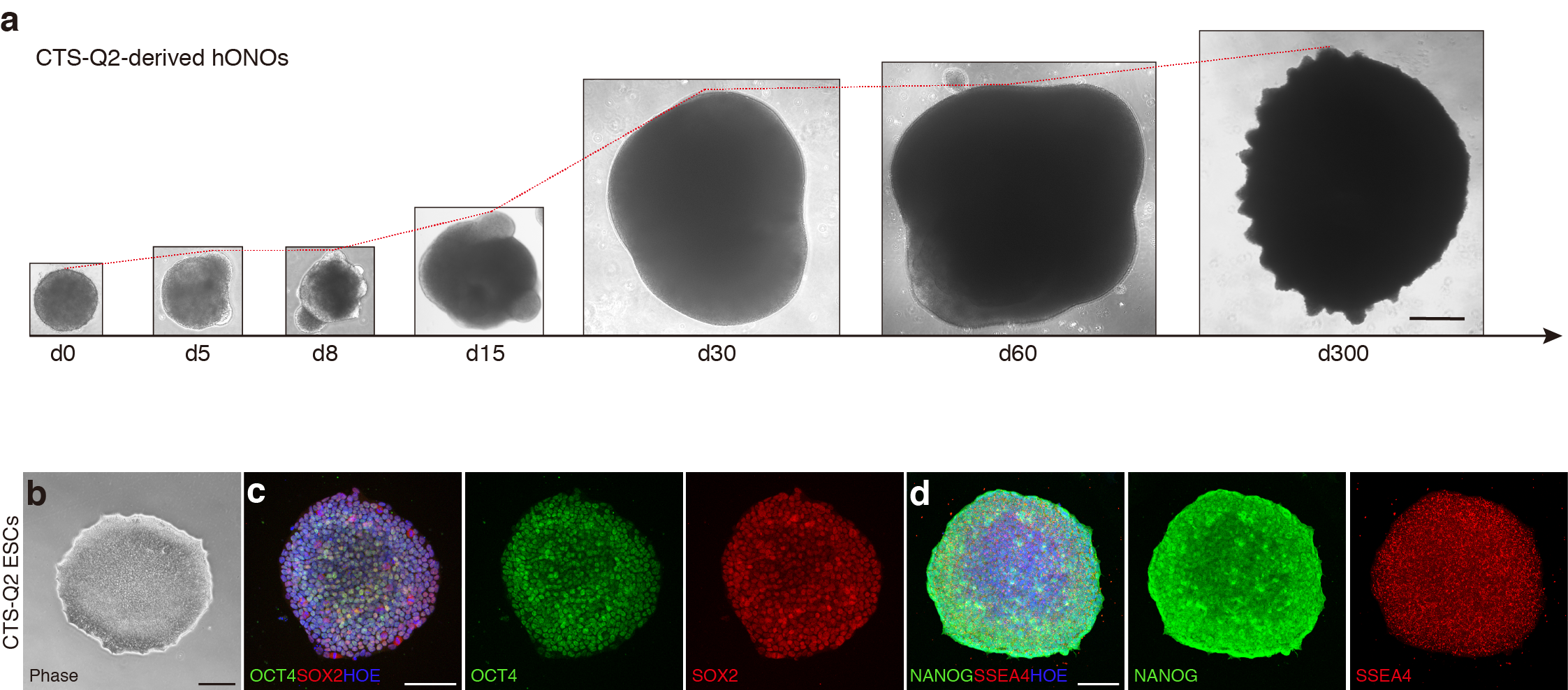

Supplement: Supplementary file 1 — FIGURE S1. Schematic view of CTS‐Q2 ESC‐derived human otic neuronal organoids. a, Phase control images showing the changes of CTS‐Q2 ESCs‐derived hONOs over time. Red dashed line showing the growth curve. b, Representative image of CTS‐Q2 ESC clones maintained on VTN‐coated surface in E8 medium. c, d, Expression of pluripotency marker genes, OCT4, SOX2, NANOG and SSEA4, in monoclonal CTS‐Q2 ESCs. Scale bars, 500 μm (a), 200 μm (b), 100 μm (c, d). [file CPR-56-e13434-s003.tif]

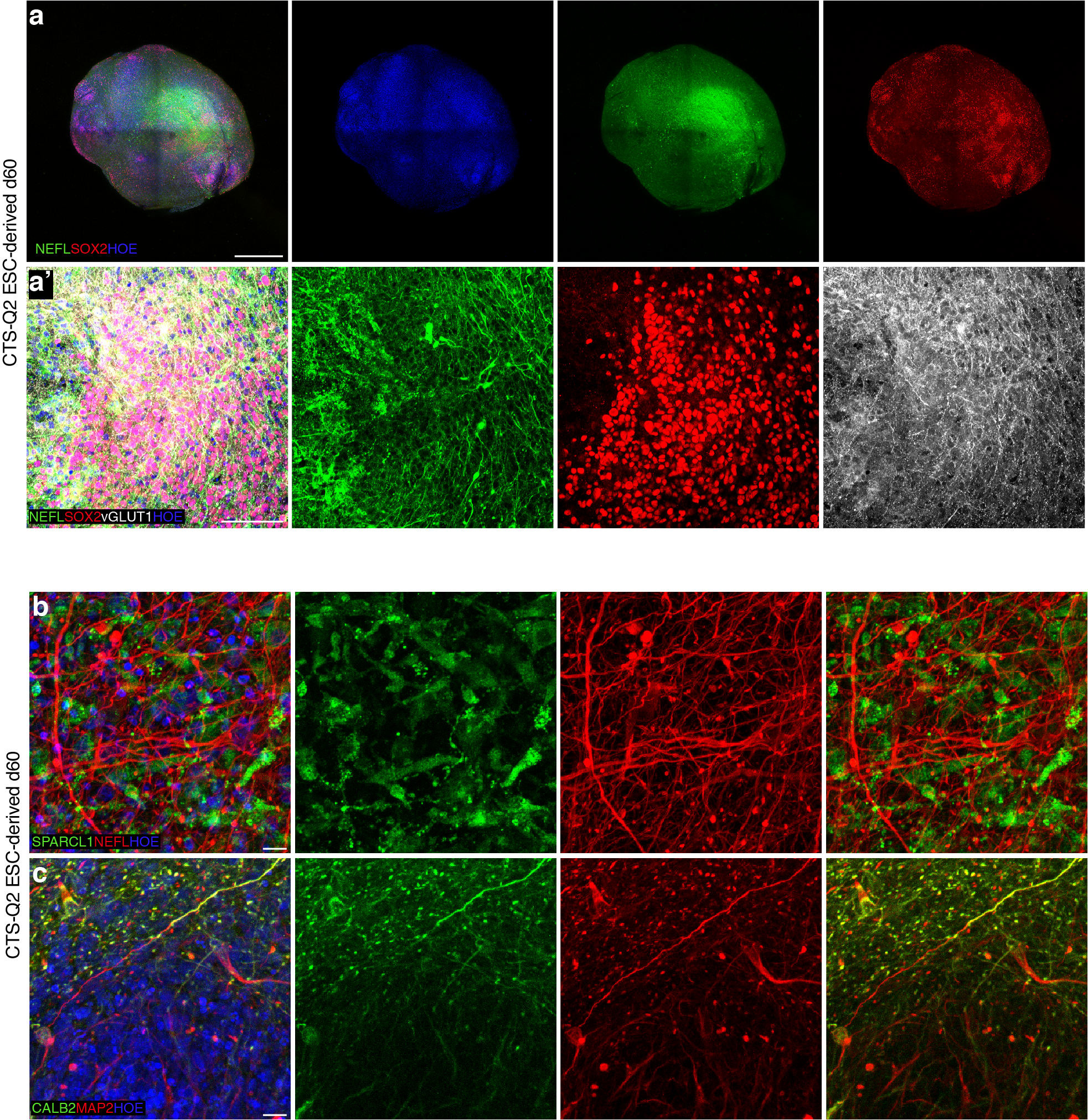

Supplement: Supplementary file 2 — FIGURE S2. SGN‐like cells and supporting cells appear in CTS‐Q2 derived hONOs at d60. a, Expression of axon marker gene (NEFL) and supporting cell marker gene (SOX2) in d60 hONOs derived from CTS‐Q2 ESCs. vGLUT1‐positive cells indicating the existence of glutamatergic neurons. b, Supporting cells (SPARCL1+) companied with axons (NEFL) in d60 hONOs. c, Expression of SGN‐specific marker gene (CALB2) and mature neuron marker gene (MAP2) in d60 hONOs. Scale bars, 500 μm (a), 100 μm (a′), 20 μm (b, c). [file CPR-56-e13434-s004.tif]

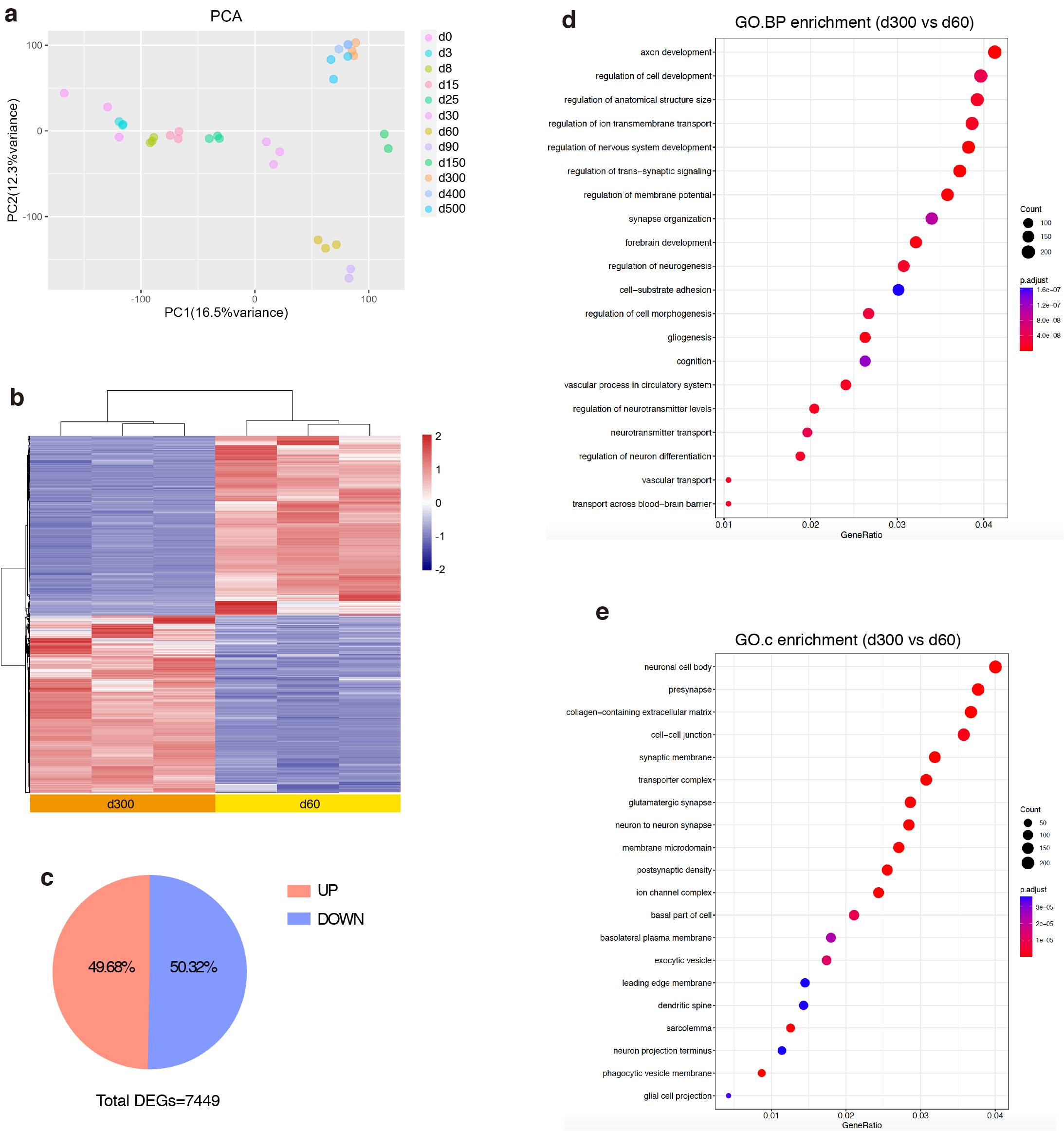

Supplement: Supplementary file 3 — FIGURE S3. Transcriptome analysis of hONOs (d0‐d500). a, Principal component analysis (PCA) depicting similarity between hONOs at the same timepoints. b, Cluster analysis of differentially expressed genes (DEGs) via comparing d300 with d60 hONOs. c, Pie chart showing up‐ and downregulated DEGs in d300 hONOs compared with d60 hONOs. d, Enriched GO.BP (d) and GO.CC (e) items in d300 hONOs compared with d60 hONOs. [file CPR-56-e13434-s007.tif]

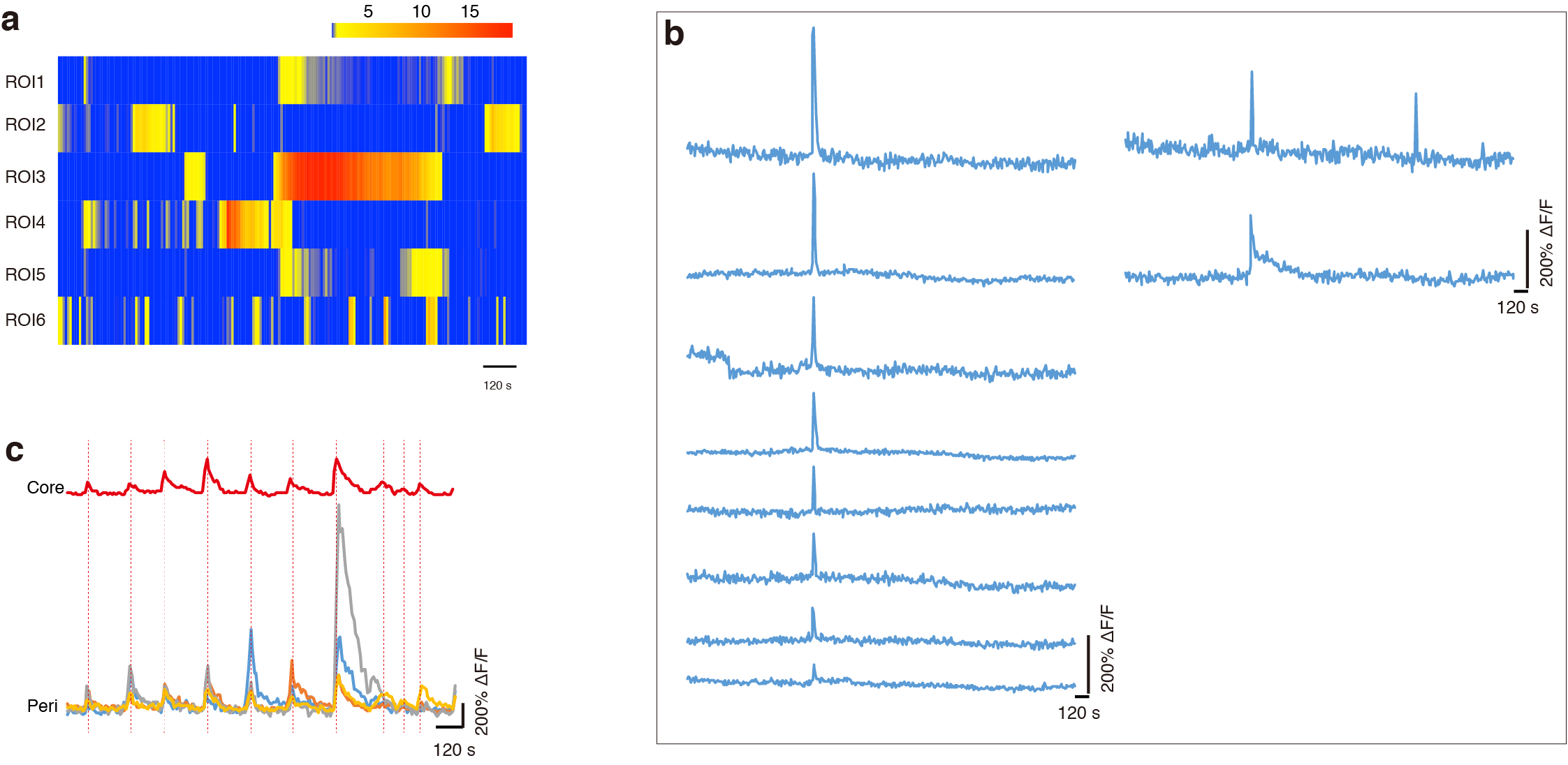

Supplement: Supplementary file 4 — FIGURE S4. Neural network in hONO at neuronal stage. a, Heat map of rosette ROIs indicated in Figure 7B. b, Calcium traces of individual ROI that contributed to a GDP‐like event in Figure 7D. c, Calcium traces of another rosette‐like population of ROIs that resembles neighbour neuron communication. [file CPR-56-e13434-s001.tif]
