# Supplementary material for: Generation of human otic neuronal organoids using pluripotent stem cells
Source: Cell Prolif. 2023 Feb 24;56(5):e13434. doi: 10.1111/cpr.13434 (PMC10212712; doi:10.1111/cpr.13434)
Supplement: Supplementary file 5 — TABLE S1. Antibodies [file CPR-56-e13434-s006.docx]

Supplementary Table 1. Antibodies

| **Antibody** | **Host** | **Supplier** | **Catalog No.** | **Dilution** |
| --- | --- | --- | --- | --- |
| Calrectinin | Goat | Millipore | AB1550 | 1:500 |
| DLX5 | Rabbit | Abcam | ab109737 | 1:200 |
| ECAD | Mouse | Abcam | ab1416 | 1:500 |
| FOXG1 | Rabbit | Abcam | ab18259 | 1:100 |
| GATA3 | Goat | R&D Systems | AF2605 | 1:200 |
| GFAP | Rat | Invitrogen | 13-0300 | 1:500 |
| MAP2 | Chicken | Novus Biologicals | NB300-213 | 1:1500 |
| MAP2 | Mouse | Abcam | ab11267 | 1:500 |
| NANOG | Rabbit | Proteintech | 14295-1-AP | 1:250 |
| NEFL | Rabbit | Abcam | ab223343 | 1:500 |
| NESTIN | Mouse | Millipore | MAB5326 | 1:250 |
| OCT4 | Mouse | BD Biosciences | 611203 | 1:200 |
| P75 | Rabbit | Cell Signaling | 8238S | 1:500 |
| PAX8 | Rabbit | Abcam | ab97477 | 1:200 |
| S100B | Rabbit | Abcam | ab52642 | 1:500 |
| SOX1 | Goat | R&D Systems | AF3369 | 1:200 |
| SOX2 | Goat | R&D Systems | AF2018 | 1:200 |
| SOX9 | Rabbit | Abcam | ab185966 | 1:250 |
| SPARCL1 | Goat | R&D Systems | AF2728 | 1:100 |
| SSEA4 | Mouse | Millipore | MAB4304 | 1:500 |
| SYP | Rabbit | Abcam | ab32127 | 1:500 |
| TRA-1-60 | Mouse | Millipore | MAB4360 | 1:500 |
| TUJ1 | Mouse | Neuromics | MO15013 | 1:500 |
| TUJ1 | Mouse | Biolegend | 801201 | 1:1000 |
| vGLUT1 | Rabbit | Invitrogen | 48-2400 | 1:200 |
